# Prognostic relevance of [18F]FDG PET/CT in triple-negative breast cancer: Results from the triple negative (TRINE)-PET trial

**DOI:** 10.1007/s00259-026-08056-1

**Published:** 2026-08-17

**Authors:** Luca Urso, Stefano Panareo, Domenico Albano, Antonella Iudicello, Luca Filippi, Francesca Borgia, Mirco Bartolomei, Priscilla Guglielmo, Francesco Bertagna, Silvia Taralli, Maria Lucia Calcagni, Angelica Mazzoletti, Mattia Bonacina, Lorenzo Fantini, Andrea Paccagnella, Carlotta Benedetta Colombo, Massimo Castellani, Alessio Annovazzi, Rosa Sciuto, Matteo Bauckneht, Federico Garrou, Demetrio Aricò, Guido Rovera, Cristina Ferrari, Alberto Miceli, Pasquale Reccia, Gianluca Cassarino, Elisa Rizza, Andrea Marongiu, Annachiara Arnone, Anna Margherita Maffione, Salvatore Ialuna, Nicola Frega, Laura Evangelista, All the TRINE-PET Investigators

**Affiliations:** 1 Department of Translational Medicine, University of Ferrara, Via Aldo Moro N.8, Ferrara, Italy; 2 Nuclear Medicine Unit, Onco-Hematology Department, University Hospital of Ferrara, Ferrara, Italy; 3 Nuclear Medicine Unit, Oncology and Hematology Department, University Hospital of Modena, Modena, Italy; 4 Nuclear Medicine, University of Brescia, Brescia, Italy; 5 Department of Biomedicine and Prevention, University Tor Vergata, Rome, Italy; 6 Department of Biomedical Sciences, Humanitas University, Pieve Emanuele, Italy; 7 Nuclear Medicine Unit, Humanitas Gavazzeni, Bergamo, Italy; 8 Nuclear Medicine Unit, Diagnostic Imaging and Radiation Oncology Department, Fondazione Policlinico Universitario A. Gemelli IRCCS, Rome, Italy; 9 Section of Nuclear Medicine, Department of Radiological Sciences and Haematology, Università Cattolica del Sacro Cuore, Rome, Italy; 10 Nuclear Medicine “Alessandra Bono”, Fondazione Poliambulanza Istituto Ospedaliero, Brescia, Italy; 11 Nuclear Medicine Department, IRCCS Istituto Romagnolo Per Lo Studio Dei Tumori “Dino Amadori” (IRST), Meldola, Italy; 12 Nuclear Medicine Service, ‘M. Bufalini’ Hospital, ‘Azienda Unità Sanitaria Locale’ Romagna, Cesena, Italy; 13 Nuclear Medicine Unit, Fondazione IRCCS Ca’ Granda Ospedale Maggiore Policlinico, Milan, Italy; 14 Nuclear Medicine Unit, IRCCS Regina Elena National Cancer Institute, Rome, Italy; 15 IRCCS Azienda Ospedaliera Metropolitana, Genova, Italy; 16 Department of Health Sciences (DISSAL), University of Genoa, Genoa, Italy; 17 Nuclear Medicine, AOU Maggiore Della Carità, Novara, Italy; 18 Nuclear Medicine Unit, Humanitas Istituto Clinico Catanese, Catania, Italy; 19 Nuclear Medicine Division, Department of Medical Sciences, AOU Città Della Salute E Della Scienza Di Torino, University of Turin, Turin, Italy; 20 Department of Nuclear Medicine, University Aldo Moro, Bari, Italy; 21 Department of Nuclear Medicine, Azienda Ospedaliero-Universitaria SS. Antonio E Biagio E Cesare Arrigo, Alessandria, Italy; 22 Unit of Nuclear Medicine and Molecular Imaging, Veneto Institute of Oncology IOV - IRCCS, Padua, Italy; 23 Nuclear Medicine Unit, Giovanni Paolo II Hospital, Ragusa, Italy; 24 Nuclear Medicine Unit, Vito Fazzi Hospital, Lecce, Italy; 25 Unit of Nuclear Medicine. Department of Medicine, Surgery and Pharmacy, University of Sassari, Sassari, Italy; 26 Department of Nuclear Medicine, Azienda Unità Sanitaria Locale-IRCCS Di Reggio Emilia, Reggio Emilia, Italy; 27 Department of Nuclear Medicine, S.M. Della Misericordia Hospital, Rovigo, Italy; 28 Nuclear Medicine Unit, Ospedali Riuniti Villa Sofia-Cervello, 90146 Palermo, Italy; 29 Nuclear Medicine Unit, Department of Health Service, AORN Ospedali Dei Colli, Naples, Italy; 30 Nuclear Medicine Unit, IRCCS Humanitas Research Hospital, Rozzano, Italy

**Keywords:** Triple negative breast cancer, PET, FDG, Prognosis, Survival

## Abstract

**Purpose:**

Triple negative breast cancer (TNBC) is an aggressive subtype, still with limited therapeutic options. [^18^F]FDG PET/CT is widely used in TNBC, but evidence regarding its prognostic value remains limited. The TRINE PET study, promoted by the Italian Association of Nuclear Medicine, aimed to evaluate the prognostic relevance and clinical impact of [^18^F]FDG PET/CT in a large real world TNBC cohort.

**Methods:**

This retrospective multicenter study included 389 TNBC patients who underwent 707 [^18^F]FDG PET/CT scans across 23 Italian Nuclear Medicine Units. Clinical settings were baseline staging (*n* = 257 patients), post neoadjuvant chemotherapy (*n* = 111 patients), suspected recurrence (*n* = 144 patients), and advanced metastatic disease (*n* = 195 patients). Progression free survival (PFS) and overall survival (OS) were assessed using Kaplan–Meier and Cox regression analyses. The influence of [^18^F]FDG PET/CT on clinical management was evaluated across all settings.

**Results:**

During a median follow up of 34 months (1–124), disease progression and death occurred in 39.3% and 28.4% of cases, respectively. Across all clinical settings, nodal and distant metastatic involvement at [^18^F]FDG PET/CT was strongly associated with worse PFS and OS (all *p* < 0.001). In multivariate analysis, distant metastases at [^18^F]FDG PET/CT resulted the strongest independent predictor of OS (HR 9.42, 95% CI 3.74–23.75) and PFS (HR 6.57, 95% CI 3.05–14.12), while Ki67 and [^18^F]FDG uptake at primary tumor did not. [^18^F]FDG PET/CT findings led to a change in clinical management in 20.8% of all scans, with the highest impact in the advanced metastatic setting (34.9%).

**Conclusion:**

In the largest real world multicenter study dedicated to TNBC, [^18^F]FDG PET/CT demonstrated robust prognostic value across all disease settings. PET detected metastatic involvement emerged as the most powerful predictor of survival; moreover, [^18^F]FDG PET/CT influenced therapeutic decision in all settings of disease, reaching one in three scans in the advanced metastatic disease. These findings support the systematic integration of [^18^F]FDG PET/CT into the diagnostic pathways of TNBC.

**Supplementary Information:**

The online version contains supplementary material available at https://doi.org/10.1007/s00259-026-08056-1.

## Introduction

Breast cancer (BC) is a highly prevalent disease. According to the most recent updates in the literature, its incidence continues to rise, with more than 2.3 million new cases reported globally each year and approximately 55,000 in Italy [1–3]. According to the St. Gallen classification, BC can be divided into five molecular subtypes based on the expression of hormone receptors (e.g. estrogen and progesterone), human epidermal growth factor receptor 2 (HER2), and the proliferative index [4]. Although the literature has long reported that the different molecular subtypes of breast cancer exhibit markedly different avidity on 2-deoxy-2-[18F]fluoro-D-glucose ([^18^F]FDG) Positron Emission Tomography/Computed Tomography (PET/CT), many studies still present results derived from mixed cohorts of BC patients [5]. The findings remain highly heterogeneous, and current clinical guidelines do not differentiate the indications for [^18^F]FDG PET/CT according to the various molecular subtypes of BC.

Triple-negative breast cancer (TNBC) remains a highly challenging disease, characterized by elevated mortality rates in patients with metastatic spread, with only modest improvements observed despite the introduction of novel treatments [6]. To ensure timely and effective treatment decisions, reliable imaging modalities play a pivotal role. In this context, [^18^F]FDG PET/CT has been increasingly adopted due to the typically high [^18^F]FDG avidity of TNBC, both in the primary tumor and in metastatic lesions [5, 7]. However, according to a recent systematic review of the literature, only preliminary data are currently available regarding the correlation between [^18^F]FDG PET/CT findings and patients’ survival outcomes [7].

Therefore, in 2023, the Italian Association of Nuclear Medicine, Molecular Imaging and Therapy (AIMN) promoted a large real-world multicenter study, named TRIple NEgative (TRINE)-PET, aimed at collecting data on the prognostic relevance and impact on clinical management of [^18^F]FDG PET/CT in TNBC patients. Herein, we present the results of the study.

## Material and methods

The TRINE-PET trial is a retrospective real-world, multicenter, observational, non-profit study. Overall, 23 Italian Nuclear Medicine Units participated in the study and collected data regarding TNBC patients undergoing [^18^F]FDG PET/CT up to December 31, 2024. The full details of the study protocol were recently published in a dedicated paper [8].

### Endpoints

The primary objective of the study is to evaluate the prognostic value of [^18^F]FDG PET/CT in patients with TNBC in four settings of disease, such as baseline, post-neoadjuvant chemotherapy (NAC), restaging for suspected recurrence and restaging in advanced metastatic setting, focusing on survival outcomes, both progression-free survival (PFS) and overall survival (OS).

The secondary objective is to assess how [^18^F]FDG PET/CT influences therapeutic decision-making across various disease settings. A change in management was defined as the occurrence of at least one of the following: (a) cancellation of surgery in patients initially considered eligible; (b) initiation of a new line of therapy; or (c) modification of the ongoing treatment, including its discontinuation and replacement with an alternative regimen.

### Harmonization and quality control

To minimize inter-center variability and ensure a consistent enrollment process, the participating centers held preliminary video-conference meetings to agree on shared criteria for patient inclusion and data collection. Specifically, only patients who had undergone [^18^F]FDG PET/CT scans of optimal diagnostic quality were included, and acquisition timing as well as administered activity were required to comply with the standard EANM recommendations for [^18^F]FDG imaging [9]. In addition, as specified in the previously published protocol [8], SUVmean values of the mediastinal blood pool and liver were collected for each scan by drawing circular volumes of interest of 1 cm and 3 cm in diameter, respectively. These reference measurements were centrally reviewed in consensus by three investigators (LE, LF, and LU) to identify any potential outliers. Since no outliers were detected, the full cohort was retained for statistical analysis, and SUVmax from the entire population was included in the final analyses.

### Statistical analysis

A descriptive statistical analysis was performed to summarize the baseline characteristics of the study population. Categorical variables were reported as absolute frequencies and percentages, whereas continuous variables were expressed as mean ± standard deviation (SD) when normally distributed, or as median with range (minimum–maximum values), when appropriate. Lesion positivity was established based on the visual interpretation documented in the clinical report of the nuclear medicine physician at each center, in order to preserve the real-world nature of the study. Physicians were unaware of the patients’ clinical outcomes when reporting.

For the analysis of the primary endpoint, time-to-event data were evaluated using Kaplan–Meier survival curves to estimate the probability of event-free survival over time. Differences between survival curves were assessed using the log-rank test. To identify potential predictors associated with the primary endpoint, both univariate and multivariate analyses were conducted using Cox proportional hazards regression models. Variables with potential clinical relevance or statistical significance in the univariate analysis were included in the multivariate model to obtain adjusted hazard ratios (HRs) with corresponding 95% confidence intervals (CIs).

For the secondary endpoint, comparisons between categorical variables were performed using the chi-square (χ^2^) test based on a 2 × 2 contingency table. When necessary, appropriate corrections were applied according to standard statistical assumptions. A two-tailed p-value of less than 0.05 was considered statistically significant. All statistical analyses were performed using MedCalc® software for Windows, version 23.3.7 (MedCalc Software Ltd., Ostend, Belgium).

## Results

A total of 707 [^18^F]FDG PET/CT scans from 389 patients with TNBC were collected. The individual contribution of each participating center is reported in Supplementary Fig. 1.

Table 1 summarizes the characteristics of the study population based on a per-scan analysis. The distribution of patients across the four clinical settings was as follows: 257 (36.4%) underwent baseline staging, 111 (15.7%) were evaluated after NAC, 144 (20.4%) underwent restaging for suspected recurrence, and 195 (27.5%) were assessed in the advanced metastatic setting. Overall, the primary tumor or a local relapse was detected in 345 scans (48.8%), whereas lymph node and distant metastases were identified in 284 (40.2%) and 181 (25.6%) scans, respectively. At baseline, PET/CT most frequently detected the primary tumor (91.1%) and regional lymph nodes (50.6%), while distant metastases were uncommon (6.6%). Following NAC, positivity for all parameters decreased, particularly for primary tumors (39.6%) and lymph node metastases at PET/CT (23.4%), with distant metastases remaining low (11.7%). In the recurrence setting, PET/CT showed lower breast recurrence detection (18.1%) but higher rates of metastatic disease, with nodal involvement in 37.5% and distant metastases in 41.7% of patients. Finally, in advanced disease, PET/CT findings were dominated by metastatic spread rather than local recurrence (21%), with distant metastases detected in 46.7% and nodal disease in 37.9% (Supplementary Table 1).

**Table 1:** Characteristics of the population for all scans and in each setting of disease

Variables	Setting of disease			
	Baseline (*n* = 257)	Post-NAC (*n* = 111)	Recurrence (*n* = 144)	Advanced disease (*n* = 195)
Distribution in percentage	36.4%	15.7%	20.4%	27.5%
Median age (range), year	51 (27–85)	48 (27–86)	55 (33–93)	54 (27–84)
Comorbidities, *n* (%)				
No	206 (80.2%)	98 (88.3%)	121 (84%)	170 (87.2%)
Yes	34 (13.2%)	7 (6.3%)	17 (11.8%)	19 (9.7%)
Unknown	17 (6.6%)	6 (5.4%)	6 (4.2%)	6 (3.1%)
Histopathological grade, *n* (%)				
G1	1 (0.4%)	0	0	0
G2	26 (10.1%)	4 (3.6%)	24 (16.7%)	24 (12.3%)
G3	213 (82.9%)	95 (85.6%)	116 (80.5%)	155 (79.5%)
Unknown	17 (6.6%)	12 (10.8%)	4 (2.8%)	16 (8.2%)
Median Ki67 (range), %	32 (10–98)	15 (10–95)	20 (10–90)	30 (10–90)
Histopathological type, *n* (%)				
NST	210 (81.7%)	91 (82%)	129 (89.6%)	167 (86.1%)
Lobular	6 (2.3%)	2 (1.8%)	4 (2.8%)	6 (3.1%)
Ductal + lobular	3 (1.2%)	0	1 (0.7%)	1 (0.5%)
Papillary	2 (0.8%)	0	0	0
Unknown	36 (14%)	18 (16.2%)	10 (6.9%)	20 (10.3%)
Family history of BC, *n* (%)				
No	157 (61.1%)	68 (61.3%)	88 (61.1%)	134 (68.7%)
Yes	82 (31.9%)	34 (30.6%)	42 (29.2%)	47 (24.1%)
Unknown	18 (7%)	9 (8.1%)	14 (9.7%)	14 (7.2%)

*BC* breast cancer, *NAC* neoadjuvant chemotherapy, *NST* no special type

### [^18^F]FDG PET/CT and survival analysis

During a median follow-up of 34 (1–124) months, disease progression and death occurred in 278 (39.3%) and 201 (28.4%) cases, respectively. As shown in Table 2, positive PET/CT findings were consistently associated with significantly higher rates of disease progression and poorer survival across all clinical settings examined (baseline, post-neoadjuvant, recurrence, and advanced metastatic). In each cohort, patients with progression and those who did not survive showed a markedly higher proportion of PET positivity compared with patients without progression and survivors. Conversely, negative PET/CT findings were more frequent among patients with favorable outcomes. All associations were statistically significant (*p* < 0.05, most *p* < 0.001).

**Table 2:** Distribution of [^18^F]FDG PET/CT results (only for lymph node and distant positivity) and outcomes

Setting, n pts	Progression, n. scans	PET/CT findings		*p-value*	Death, n scans	PET/CT findings		*p-value*
		Negative	Positive			Negative	Positive	
Baseline, 257	Yes, 64	15 (23%)	49 (77%)	< *0.001*	Yes, 32	3 (9%)	29 (91%)	< *0.001*
	No, 193	109 (56%)	84 (44%)		No, 225	121 (54%)	104 (46%)	
Post-Neoadjuvant setting, 111	Yes, 33	9 (26%)	24 (74%)	= *0.03*	Yes, 22	5 (23%)	17 (77%)	= *0.03*
	No, 78	40 (51%)	38 (49%)		No, 89	44 (44%)	45 (56%)	
Recurrence of disease, 144	Yes, 70	11 (16%)	59 (84%)	< *0.001*	Yes, 55	7 (13%)	48 (87%)	< *0.001*
	No, 74	39 (53%)	35 (47%)		No, 89	43 (48%)	46 (52%)	
Advanced metastatic setting, 195	Yes, 111	23 (21%)	88 (79%)	< *0.001*	No, 92	16 (17.4%)	76 (82.6%)	< *0.001*
	No, 84	48 (57%)	36 (43%)		Yes, 103	55 (53%)	48 (47%)	

**Fig. 1:**
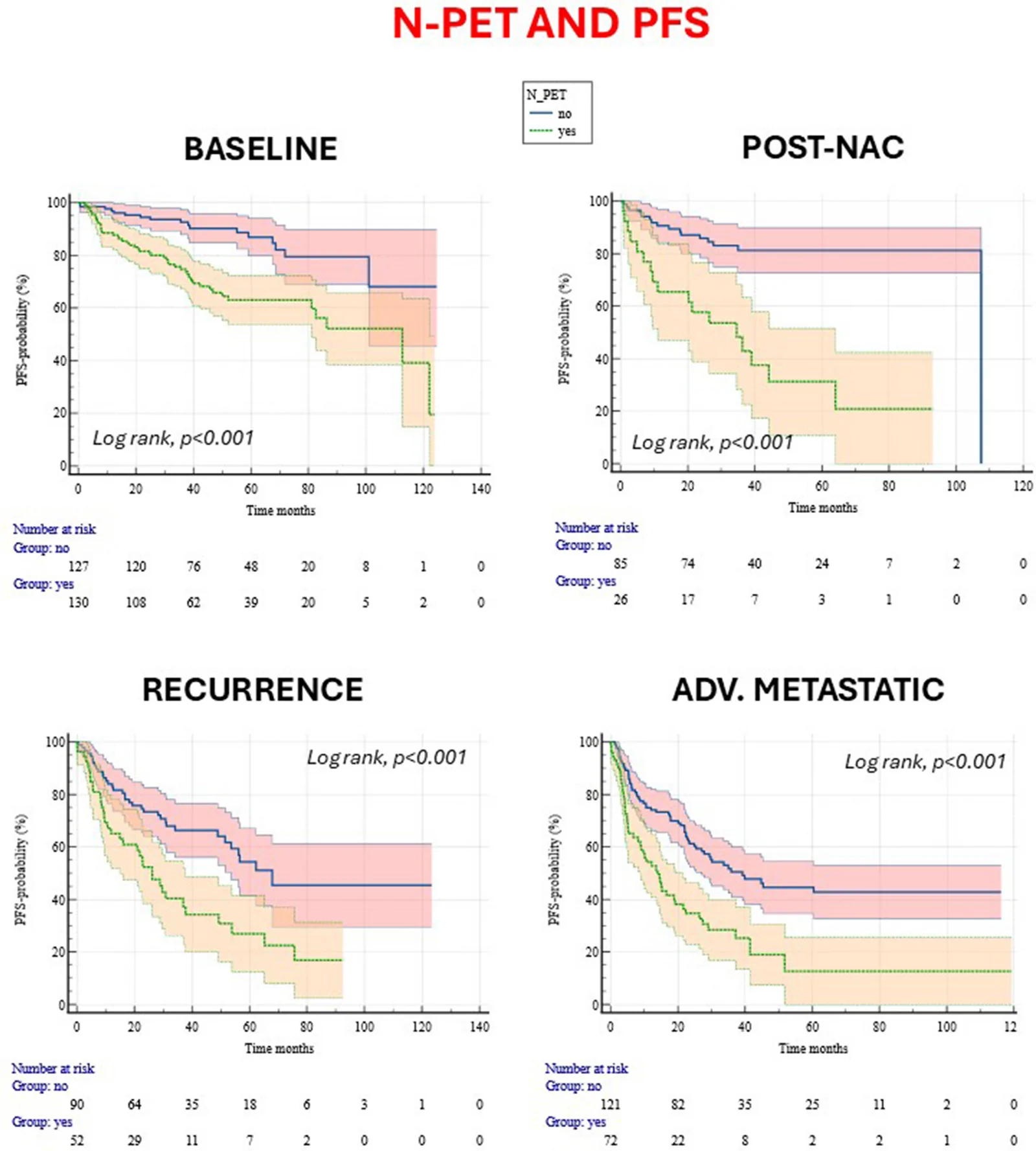
N-PET and progression-free survival (PFS) across clinical settings. Kaplan–Meier curves showing PFS according to nodal status on [^18^F]FDG PET/CT (N-PET positive vs negative) in four clinical scenarios: baseline staging, post-NAC, recurrence, and advanced metastatic disease. In all panels, PET-positive nodal involvement was associated with significantly shorter PFS (log-rank *p* < 0.001). Numbers at risk are reported below each plot

**Fig. 2:**
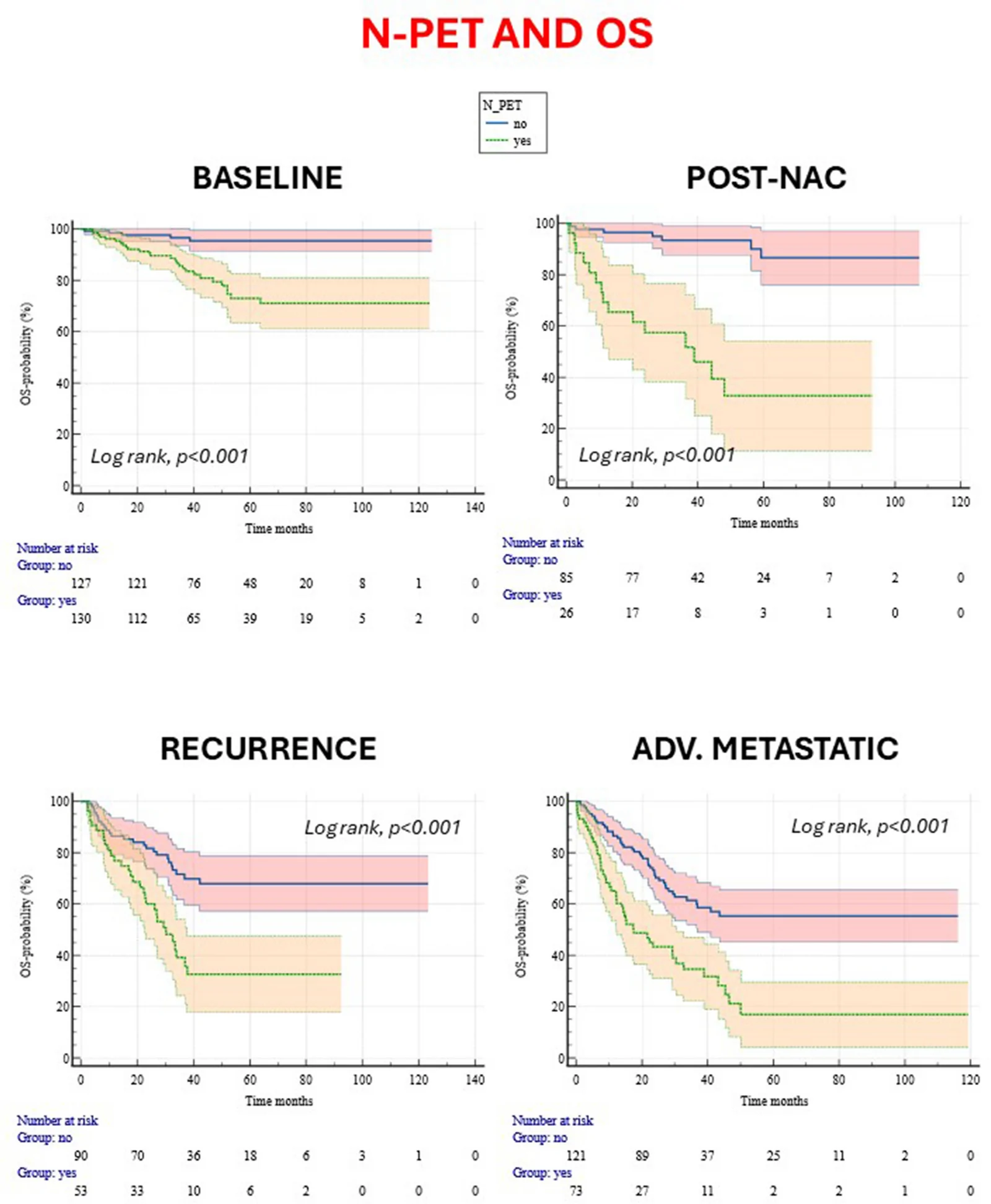
N-PET and overall survival (OS) across clinical settings. Kaplan–Meier curves showing OS according to nodal status on [^18^F]FDG PET/CT (N-PET positive vs negative) in baseline, post-NAC, recurrence, and advanced metastatic settings. PET-positive nodal involvement consistently correlated with worse OS (log-rank *p* < 0.001). Numbers at risk are reported below each plot

In all four clinical settings, the presence of lymph node metastases at [^18^F]FDG PET/CT was consistently associated with worse outcomes (Figs. 1 and 2). Specifically, lymph node involvement correlated with a significantly higher risk of disease progression (all *p* < 0.001 for baseline, post-NAC, suspected recurrence, and advanced metastatic setting). Similarly, lymph node metastases were also linked to a significantly increased risk of death across all settings (all *p* < 0.001).

**Fig. 3:**
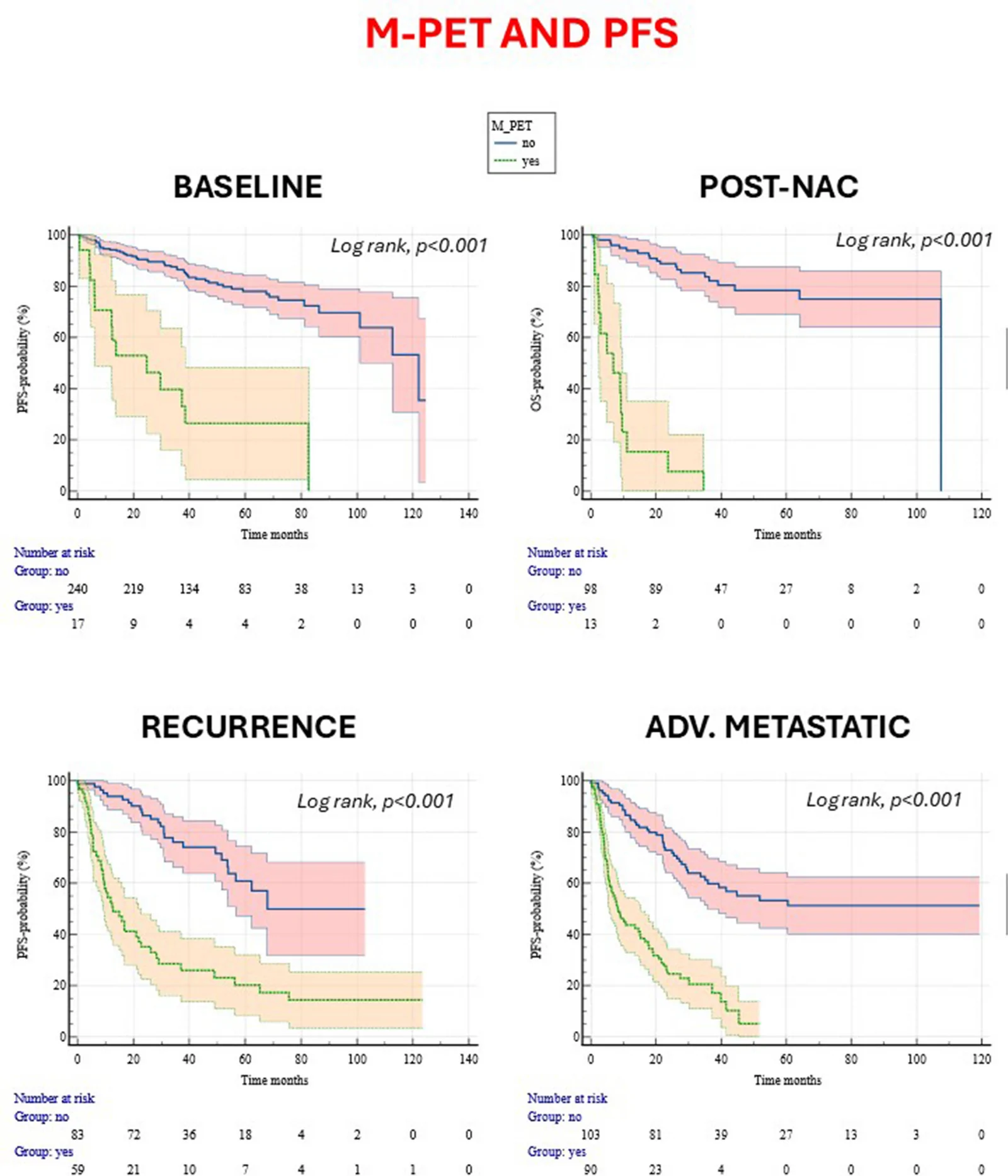
M-PET and progression-free survival (PFS) across clinical settings. Kaplan–Meier curves showing PFS according to metastatic status on [^18^F]FDG PET/CT (M-PET positive vs negative) in baseline, post-NAC, recurrence, and advanced metastatic settings. PET-positive distant metastases were associated with markedly reduced PFS in all scenarios (log-rank *p* < 0.001). Numbers at risk are reported below each plot

**Fig. 4:**
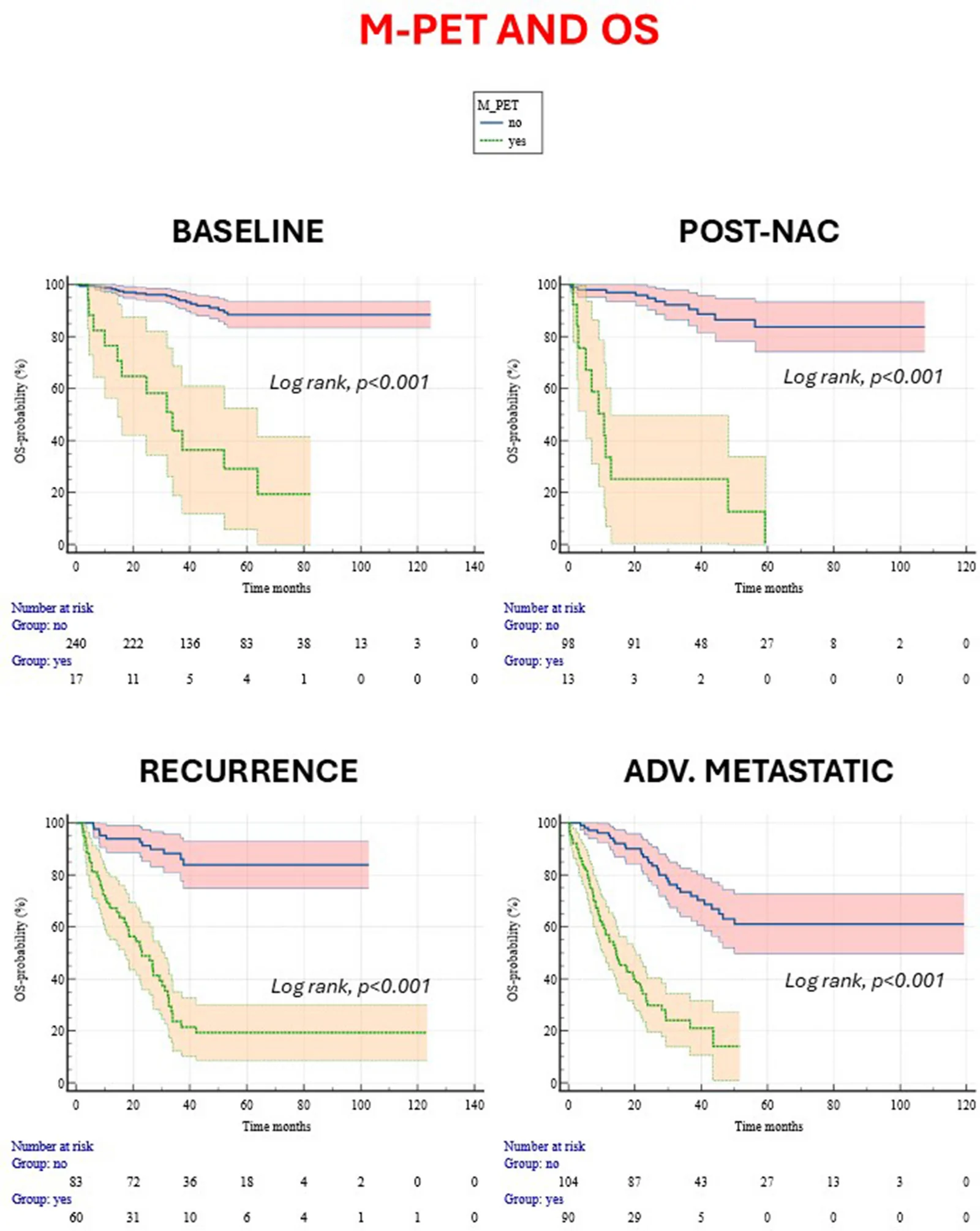
M-PET and overall survival (OS) across clinical settings. Kaplan–Meier curves showing OS according to metastatic status on [^18^F]FDG PET/CT (M-PET positive vs negative) in baseline, post-NAC, recurrence, and advanced metastatic settings. PET-positive metastatic disease was associated with significantly worse OS across all clinical scenarios (log-rank *p* < 0.001). Numbers at risk are reported below each plot

Similarly, a comparable prognostic stratification emerged in the presence of distant metastases on [^18^F]FDG PET/CT (Figs. 3 and 4). Across all four clinical settings, distant metastatic involvement was strongly associated with an increased risk of both disease progression (all *p* < 0.001) and death (all *p* < 0.001).

PET positivity was consistently associated with inferior PFS across all clinical settings, with stronger effects observed for metastatic than nodal findings. At baseline, PET-detected lymph node (N-PET) and metastatic disease (M-PET) were associated with increased risk of progression (HR 2.92, 95% CI 1.68–5.10 and HR 6.32, 95% CI 3.41–11.73, respectively). Similar associations were observed in the post-NAC setting, in advanced disease, and at recurrence. In contrast, PET positivity showed limited prognostic value for OS. N-PET status was not significantly associated with OS in any setting. Positive M-PET findings were associated with worse OS only in advanced disease. Overall, metastatic PET positivity emerged as the strongest predictor of disease progression, particularly in the post-NAC setting (Table 3).

**Table 3:** [^18^F]FDG-PET prognostic value across all clinical settings

Clinical setting	PET site	Outcome	N pts	Median value (negative scan)	Median value (positive scan)	12-mo survival (negative scan)	12-mo survival (positive scan)	HR (positive vs negative)	95% CI low	95% CI high
Baseline	N_PET	PFS	257	inf	112,67	0,97	0,88	2,923	1,677	5,095
Baseline	N_PET	OS	257	48,97	51,27	0,98	0,96	0,908	0,698	1,182
Baseline	M_PET	PFS	257	122,03	24,73	0,94	0,71	6,324	3,409	11,730
Baseline	M_PET	OS	257	48,97	63,90	0,97	1,00	0,717	0,295	1,744
post NAC	N_PET	PFS	111	107,47	34,60	0,91	0,65	4,790	2,387	9,610
post NAC	N_PET	OS	111	42,67	50,47	0,98	1,00	0,820	0,434	1,549
post NAC	M_PET	PFS	111	107,47	6,87	0,94	0,15	20,124	8,969	45,151
post NAC	M_PET	OS	111	43,70	inf	0,99	0,92	1,013	0,247	4,159
Recurrence	N_PET	PFS	144	67,90	26,17	0,84	0,65	2,236	1,396	3,582
Recurrence	N_PET	OS	144	46,37	49,30	0,96	0,92	1,066	0,664	1,713
Recurrence	M_PET	PFS	144	67,90	12,67	0,94	0,53	4,362	2,663	7,144
Recurrence	M_PET	OS	144	40,07	65,23	0,95	0,94	0,486	0,276	0,856
Advanced disease	N_PET	PFS	195	39,27	13,97	0,74	0,54	2,231	1,527	3,261
Advanced disease	N_PET	OS	195	41,87	37,20	0,95	0,84	1,223	0,781	1,915
Advanced disease	M_PET	PFS	195	inf	8,40	0,86	0,44	4,202	2,814	6,277
Advanced disease	M_PET	OS	195	50,00	30,23	0,95	0,86	2,905	1,771	4,764

*NAC* neoadjuvant chemotherapy, *N* lymph node, *M* metastasis, *PFS* progression free survival, *OS* overall survival, *HR* hazard ratio, *CI* confidence interval

### Univariable and multivariable analyses

Univariable and multivariable Cox regression analyses across the different clinical settings consistently identified PET-detected metastatic disease (M-PET positivity) as the strongest predictor of progression-free survival (PFS) (Supplementary Table 2). At baseline, both N-PET positivity (HR 2.14, 95% CI 1.16–3.94) and M-PET positivity (HR 5.62, 95% CI 2.81–11.23) independently predicted disease progression. Following neoadjuvant chemotherapy (post-NAC), only M-PET positivity remained independently associated with shorter PFS (HR 11.41, 95% CI 3.85–33.75). Similarly, in the recurrence setting, M-PET positivity (HR 3.97, 95% CI 2.29–6.87), together with age, independently predicted progression, whereas N-PET positivity lost significance after adjustment. In patients with advanced disease, several factors retained prognostic value for PFS, including age, oncologic comorbidities, T-PET positivity, N-PET positivity, and M-PET positivity (HR 2.91, 95% CI 1.86–4.56), with metastatic PET involvement again showing the strongest association with disease progression. In contrast, PET findings were generally not associated with OS in the baseline, post-NAC, and recurrence settings. The only exception was the advanced disease cohort, in which M-PET positivity independently predicted worse OS (HR 1.81, 95% CI 1.02–3.21), together with older age and T-PET positivity.

In the entire population, independent of the clinical setting (adjusted model), a multivariate Cox proportional hazards analysis was performed including age, Ki67, SUVmax of the primary tumor, nodal status at PET/CT and metastatic status at PET/CT. The presence of PET-detected distant metastases was the strongest independent prognostic factor for OS (HR 9.42, 95% CI 3.74–23.75, *p* < 0.001). PET-positive nodal involvement was also independently associated with worse OS (HR 6.35, 95% CI 2.05–19.69, *p* = 0.001). Age showed a borderline association with OS (HR 1.03 per year increase, 95% CI 0.99–1.06, *p* = 0.061), while Ki67 and SUVmax of the primary tumor were not significantly associated with OS. Similarly, a positive metastatic status at PET independently predicted shorter PFS (HR 6.57, 95% CI 3.05–14.12, *p* < 0.001), as did positive-PET lymph node disease (HR 2.66, 95% CI 1.45–4.88, *p* = 0.002). Age was modestly but significantly associated with PFS (HR 1.02 per year, 95% CI 1.00–1.04, *p* = 0.034). Ki67 and SUVmax of the primary tumor were not independently associated with PFS (Table 4). Nevertheless, given the limited number of events in some clinical-setting subgroups, subgroup-specific multivariable estimates should be interpreted with caution.

**Table 4:** Multivariable analysis for OS and PFS, adjusted for all settings of disease

Variable	OS HR (95% CI)	*p-value*	PFS HR (95% CI)	*p-value*
Age (per year)	1.03 (0.99–1.06)	*0.061*	1.02 (1.00–1.04)	*0.034*
Ki67	1.01 (1.00–1.03)	*0.136*	0.99 (0.98–1.01)	*0.318*
T_SUVmax	0.97 (0.91–1.03)	*0.317*	0.97 (0.93–1.01)	*0.130*
N_PET (N + vs. N-)	6.35 (2.05–19.69)	*0.001*	2.66 (1.45–4.88)	*0.002*
M_ PET (M + vs. M-)	9.42 (3.74–23.75)	< *0.001*	6.57 (3.05–14.12)	< *0.001*

*OS* overall survival, *HR* hazard ratio, *CI* confidence interval, *PFS* progression free survival, *T* primary tumor or breast recurrence; *SUVmax* maximum standardized uptake value, *N* Nodal metastasis, *M* distant metastasis, *N +* positive nodal metastasis, *M +*positive distant metastasis

### [^18^F]FDG PET/CT impact on change of management

**Fig. 5:**
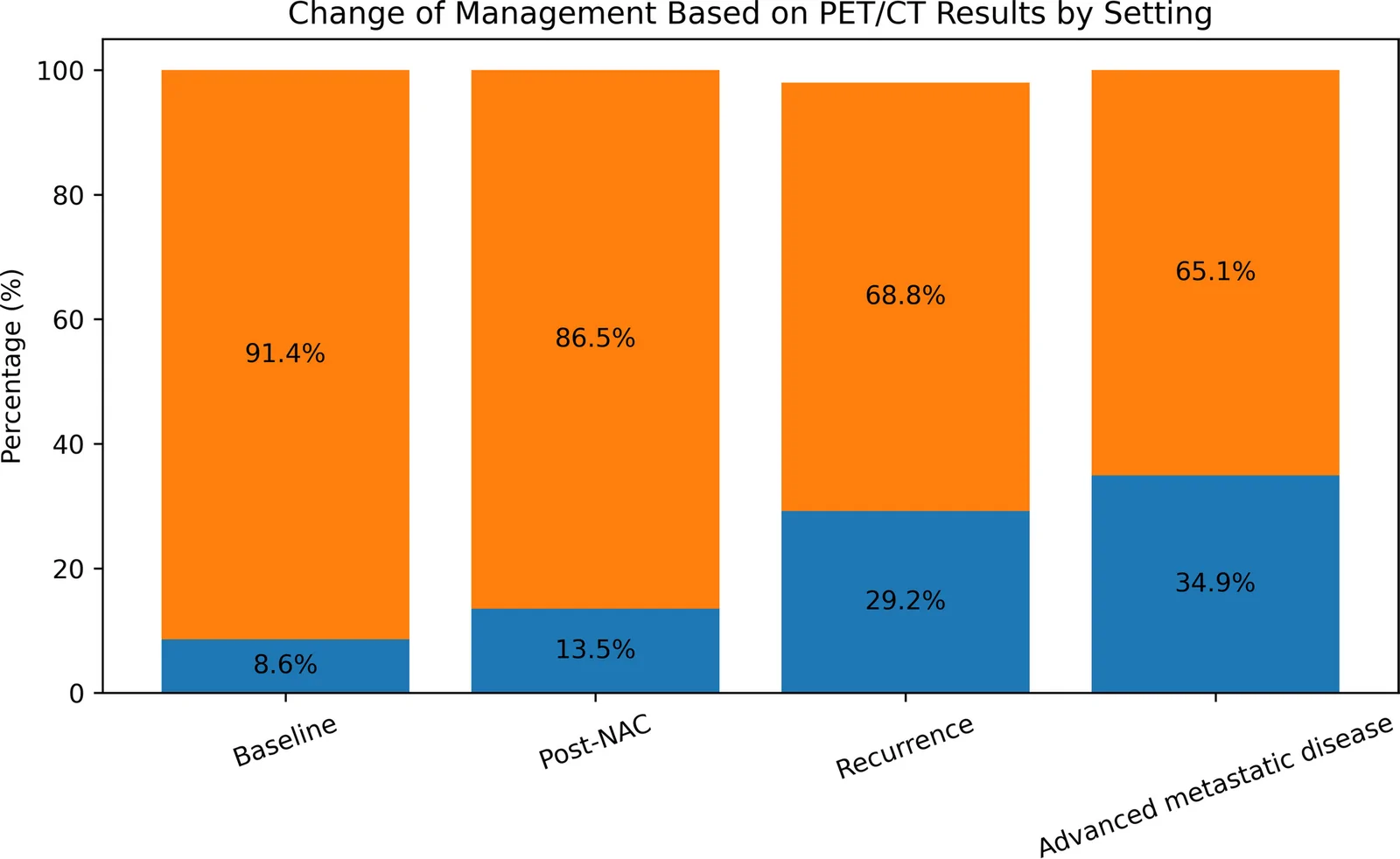
Change of management based on [^18^F]FDG PET/CT results across clinical settings. Stacked bar chart showing the proportion of patients whose management changed following [^18^F]FDG PET/CT in four clinical scenarios: baseline staging, post-NAC, recurrence, and advanced metastatic disease. The percentage of management changes increased progressively with disease stage, reaching the highest impact in the metastatic setting (34.9%). Percentages for each category are displayed within the bars

Overall, a change in clinical management was documented after 147 [^18^F]FDG PET/CT scans (20.8%). The impact of [^18^F]FDG PET/CT on determining changes in clinical management was significant across all clinical settings investigated, although heterogeneous (Fig. 5; Table S3). An increasing proportion of management changes was observed as patients progressed to more advanced stages of disease. In particular, the highest percentage was observed in the advanced metastatic setting (34.9%). At baseline, [^18^F]FDG PET/CT findings led to a change in management in 8.6% of patients (*n* = 22/257). Management changes were significantly associated with both lymph node metastasis (73% vs. 49% in those without change, *p* < 0.05) and distant metastasis (36% vs. 4%, *P* < 0.001). Following neoadjuvant chemotherapy (post-NAC), management was altered in 13.5% of cases (*n* = 15/111). While lymph node metastasis rates did not differ significantly between groups (27% vs. 23%, *p* = NS), distant metastasis remained strongly associated with management change (40% vs. 7%, *p* < 0.001). At recurrence, management changes occurred in 29.2% of patients (*n* = 42/144). These changes were significantly associated with lymph node metastasis (50% vs. 32%, *p* < 0.05), whereas the presence of distant metastasis was not significantly different between groups (52% vs. 37%, *p* = NS). In advanced metastatic disease, [^18^F]FDG PET/CT influenced management decisions in 34.9% of patients (*n* = 68/195). Both lymph node metastasis (63% vs. 24%, *p* < 0.001) and distant metastasis (75% vs. 31%, *p* < 0.001) were significantly more frequent among patients whose management was changed.

## Discussion

This large multicenter study provides robust real-world evidence supporting the pivotal role of [^18^F]FDG PET/CT in the management of TNBC. Across all clinical settings evaluated—baseline staging, post-neoadjuvant chemotherapy, restaging for suspected recurrence, and advanced metastatic disease—[^18^F]FDG PET/CT consistently demonstrated a strong prognostic relevance. This aligns with previous evidence showing that TNBC exhibits markedly elevated glucose metabolism compared with other BC subtypes [5, 7]. The high metabolic activity typical of TNBC translates into excellent detectability on PET/CT, making it a valuable tool throughout the patient’s natural history of disease and stratifying patients according to their prognosis.

At baseline, [^18^F]FDG PET/CT identified the primary tumor in more than 90% of cases, confirming its high detection rate not only for nodal and distant staging but also for the primary tumor assessment. This is particularly relevant given that current guidelines do not differentiate [^18^F]FDG PET/CT indications based on molecular subtype [10], despite accumulating evidence that TNBC benefits disproportionately from metabolic imaging due to its high [^18^F]FDG avidity [5]. The ability to accurately characterize the primary tumor, detect nodal involvement, and identify distant metastases supports the integration of [^18^F]FDG PET/CT into the initial staging of TNBC [11]. After NAC, [^18^F]FDG PET/CT emerges as a pivotal imaging modality, capable of extending the field of view beyond breast MRI, which remains the gold standard for assessing local response to treatment [12]. According to our data, [^18^F]FDG PET/CT findings can identify patients at higher risk of progression and death during follow-up, thereby informing treatment escalation or de-escalation strategies [13, 14]. In patients undergoing restaging for suspected recurrence, [^18^F]FDG PET/CT demonstrated an excellent detection rate in identifying the site of relapse, including local recurrence, nodal involvement, and distant metastases. This is particularly important in TNBC, where recurrences tend to occur early and with aggressive patterns [6]. The ability of [^18^F]FDG PET/CT to rapidly and accurately localize disease allows timely therapeutic intervention, which is crucial in a subtype with limited therapeutic windows, thus reinforcing its prognostic role.

One of the most compelling findings of the TRINE-PET study is the consistent association between [^18^F]FDG PET positivity—both nodal (N +) and metastatic (M +)—and survival outcomes across all clinical settings. In every phase of disease, the presence of PET-detected N + or M + disease correlated significantly with both PFS and OS. This uniform prognostic signal suggests that [^18^F]FDG PET/CT findings function as a reliable prognostic stratified tool in TNBC, capturing clinically meaningful disease burden.

Across clinical settings, PET-detected metastatic disease emerged as the most consistent predictor of disease progression, retaining independent prognostic significance in baseline, post-NAC, recurrent, and advanced disease cohorts. These findings suggest that PET positivity may primarily reflect the risk of progression rather than mortality, as significant associations with OS were generally not observed. This may be partly attributable to the limited number of death events, the availability of effective subsequent therapies, and the relatively favorable survival outcomes observed in several cohorts. Notably, the prognostic impact of M-PET positivity was particularly pronounced following neoadjuvant chemotherapy, supporting the potential value of post-treatment PET assessment for identifying patients at increased risk of early relapse. In the recurrence setting, the unexpected inverse association between M-PET positivity and OS should be interpreted cautiously, as it may reflect residual confounding, treatment-related factors, or limited statistical power. In contrast, in advanced disease, PET findings, particularly M-PET positivity, were associated with both PFS and OS, suggesting that PET-derived measures may provide incremental prognostic information beyond conventional clinical factors and could contribute to patient risk stratification.

Conversely, SUVmax of the primary tumor and Ki67 did not show independent prognostic value, consistent with prior evidence that disease extent, rather than primary tumor biology, is the dominant determinant of prognosis in TNBC [5, 15]. Although expected, our experience is the first to report the prognostic significance of [^18^F]FDG PET/CT in a large-cohort real-world experience. A few papers reported the prognostic relevance of [^18^F]FDG PET/CT in luminal breast cancer [16] and also in TNBC [17–20]. Kim et al. [20] reported that the metabolic and volumetric parameters of lymph node metastases at baseline were significant predictors of postoperative recurrence. Although our study did not specifically evaluate metabolic or volumetric PET parameters, our findings confirm the prognostic relevance of detecting lymph node metastases at baseline, demonstrating a clear association with both reduced PFS and OS. Similar results have also been reported by Bouron et al. [17]. Again, in the baseline setting, the authors report that lymph node involvement detected by [^18^F]FDG PET/CT was associated with lower PFS at multivariate analysis, in consistence with our data. However, they observed that only volumetric PET parameters at baseline were prognostically relevant for predicting OS. In contrast, our data demonstrated a strong association between metastatic disease detected at baseline [^18^F]FDG PET/CT and OS, likely attributable to the substantially larger sample size of our cohort (257 vs. 111 patients, respectively in our and Bouron’s study; [17]). Finally, in the metastatic setting, Marinelli et al. [19] found high metabolic tumor volume (MTV) as a strong predictor of shorter OS. Again, although quantitative PET parameters were beyond the scope of our analysis due to the large number of participating institutions and the resulting variability in acquisition protocols, we found a strong correlation between [^18^F]FDG PET/CT–detected lymph node and distant metastases and both PFS and OS. These findings suggest that the simple presence versus absence of PET-positive disease already provides substantial prognostic information, without the need for semi-quantitative or volumetric measurements. However, the role of quantitative PET parameters in BC warrants further investigation using standardized methodologies or software capable of generating reproducible results, in the same way as prostate cancer [18, 21].

Overall, [^18^F]FDG PET/CT-driven management changes were most strongly associated with the detection of distant metastases, particularly in the baseline, post-NAC, and advanced disease settings, while lymph node metastasis showed variable impact across clinical scenarios. A clinically meaningful change in management occurred after 20.8% of [^18^F]FDG PET/CT scans. This confirms that [^18^F]FDG PET/CT not only provides diagnostic and prognostic information but also directly influences therapeutic decision-making in 1 patient out of 5. Changes included cancellation of planned surgery at baseline and after NAC—primarily due to the unexpected detection of distant metastases—while in more advanced settings, the identification of metabolically active disease led to the initiation of new systemic therapies, or the discontinuation and replacement of ineffective treatments, thereby actively guiding oncologists in critical therapeutic choices. Of note, the highest impact in changing management was observed in the metastatic setting (34.9%), with a progressive percentage increase from baseline to metastatic disease, following the natural history of TNBC progression. This stepwise rise reflects the increasing clinical complexity and therapeutic vulnerability of patients as the disease advances: while early-stage [^18^F]FDG PET/CT findings may refine staging or modify surgical planning, in the metastatic phase [^18^F]FDG PET/CT becomes a decisive tool, frequently uncovering additional sites of disease and prompting immediate therapeutic adjustments in more than 1 patient out of 3. These findings align with previous smaller studies reporting that [^18^F]FDG PET/CT frequently alters management in high-risk BC patients [22–26], but TRINE-PET is the first to quantify this effect specifically in TNBC on a national scale.

The strengths of this study include its large sample size, multicenter design, and real-world nature, which enhance generalizability. The inclusion of four distinct clinical settings provides a comprehensive overview of the role of [^18^F]FDG PET/CT across the entire disease continuum. Limitations include the retrospective design and the lack of standardized PET acquisition protocols across centers, although this variability reflects real-world practice. Additionally, advanced PET metrics such as MTV or total lesion glycolysis (TLG) were not evaluated and may warrant investigation in future prospective studies. An additional limitation is that the specific nature of PET/CT-driven management changes (e.g., treatment escalation, treatment modification, or cancellation of planned interventions) was not systematically captured, limiting a more detailed assessment of the clinical impact of PET/CT findings.

The TRINE-PET study provides compelling evidence that [^18^F]FDG PET/CT should be considered a central imaging modality in TNBC. Its ability to stratify prognosis and influence clinical management supports a more systematic integration into diagnostic and therapeutic pathways. Future research should explore the integration of PET-based biomarkers with molecular and genomic data, as well as the potential role of radiomics and artificial intelligence to further refine prognostic models and guide personalized treatment strategies in patients with TNBC.

## Conclusion

In the largest real-world multicenter study dedicated to TNBC, [^18^F]FDG PET/CT demonstrated excellent prognostic value, and a substantial impact on clinical management across all disease settings. PET-detected nodal and metastatic involvement emerged as the most powerful predictors of survival, underscoring the importance of metabolic imaging in this aggressive BC subtype. These findings support the broader adoption of [^18^F]FDG PET/CT as a key component of the clinical pathway in TNBC.

## Supplementary Information

Below is the link to the electronic supplementary material.

Supplementary file1 (DOCX 31.9 KB)

Supplementary file2 (JPG 79.1 KB)

## Data Availability

The datasets generated during and/or analysed during the current study are available from the corresponding author on reasonable request.
