# Supplementary material for: Prognostic relevance of [18F]FDG PET/CT in triple-negative breast cancer: Results from the triple negative (TRINE)-PET trial
Source: Eur J Nucl Med Mol Imaging. 2026 Aug 17;53(13):8333–46. doi: 10.1007/s00259-026-08056-1 (PMC13727922; doi:10.1007/s00259-026-08056-1)
Supplement: Supplementary file 1 [file 259_2026_8056_MOESM1_ESM.docx]

**Indexed Authors full list**

| **Name** | **Affiliation** |
| --- | --- |
| Maria Luisa De Rimini | Nuclear Medicine Unit, Department of Health Service, AORN Ospedali dei Colli, Naples, Italy |
| Gian Mauro Sacchetti | Nuclear Medicine, AOU Maggiore della Carità, Novara, Italy |
| Angela Spanu | Unit of Nuclear Medicine. Department of Medicine, Surgery and Pharmacy, University of Sassari, Sassari, Italy |
| Alfredo Muni | Department of Nuclear Medicine, Azienda Ospedaliero-Universitaria SS. Antonio e Biagio e Cesare Arrigo, Alessandria, Italy |
| Natale Quartuccio | Nuclear Medicine Unit, Ospedali Riuniti Villa Sofia-Cervello, 90146 Palermo, Italy |
| Michelangelo Bambaci | Nuclear Medicine Unit, Humanitas Istituto Clinico Catanese, Catania, Italy |
| Stefania Sorbello | Nuclear Medicine Unit, Giovanni Paolo II Hospital, Ragusa, Italy |
| Lucia Setti | Nuclear Medicine Unit, Humanitas Gavazzeni, Bergamo, Italy |
| Luca Sofia | IRCCS Azienda Ospedaliera Metropolitana, Genova, Italy |
| Maria Luce Mangia | Nuclear Medicine Division, Department of Medical Sciences, AOU Città della Salute e della Scienza di Torino, University of Turin, Turin, Italy |
| Lorenzo Cecconetto | Medical Oncology, Breast & GYN Unit, IRCCS Istituto Romagnolo per lo Studio dei Tumori (IRST) "Dino Amadori", Meldola, Italy |
| Lea Cuppari | Unit of Nuclear Medicine and Molecular Imaging, Veneto Institute of Oncology IOV - IRCCS, Padua, Italy |
| Naima Ortolan | Department of Nuclear Medicine, S.M. della Misericordia Hospital, Rovigo, Italy |
| Davide Donner | Nuclear Medicine Unit, APSS della Provincia Autonoma di Trento, Santa Chiara Hospital, Trento, Italy |

**Table S1.** [^18^F]FDG PET/CT results in all settings and for each of them

| Variables | **All scans (n=707)** | **Setting of disease** | | | |
| --- | --- | --- | --- | --- | --- |
|  |  | Baseline  (n=257 pts) | Post-NAC  (n=111 pts) | Recurrence  (n=144 pts) | Advanced disease  (n=195 pts) |
| **T_PET, n (%)**  No  Yes | 362 (51.2%)  345 (48.8%) | 23 (8.9%)  234 (91.1%) | 67 (60.4%)  44 (39.6%) | 118 (81.9%)  26 (18.1%) | 154 (79%)  41 (21%) |
| **N_PET, n (%)**  No  Yes | 423 (59.8%)  284 (40.2%) | 127 (49.4%)  130 (50.6%) | 85 (76.6%)  26 (23.4%) | 90 (62.5%)  54 (37.5%) | 121 (62.1%)  74 (37.9%) |
| **M_PET, n (%)**  No  Yes | 526 (74.4%)  181 (25.6%) | 240 (93.4%)  17 (6.6%) | 98 (88.3%)  13 (11.7%) | 84 (58.3%)  60 (41.7%) | 104 (53.3%)  91 (46.7%) |

T=primary tumor or breast recurrence; N=nodal involvement; M=metastatic involvement; pts=patients

**Supplementary Table 2.**

| **Outcome** | **Analysis** | **Variable** | **Baseline** | | | | | **Post-NAC** | | | | **Recurrence** | | | | **Advanced disease** | | | |
| --- | --- | --- | --- | --- | --- | --- | --- | --- | --- | --- | --- | --- | --- | --- | --- | --- | --- | --- | --- |
|  |  |  | **HR** | **95% CI**  **Low** | **95% CI**  **High** | ***p-value*** | **HR** | | **95% CI Low** | **95% CI High** | ***p-value*** | **HR** | **95% CI Low** | **95% CI High** | ***p-value*** | **HR** | **95% CI Low** | **95% CI High** | ***p-value*** |
| PFS | Univariable | Age | 1,017 | 0,997 | 1,037 | *0,089* | 1,018 | | 0,988 | 1,049 | *0,239* | 1,029 | 1,011 | 1,048 | *<0,005* | 1,028 | 1,013 | 1,043 | *<0,001* |
|  |  | Comorbidities | 0,748 | 0,321 | 1,744 | *0,502* | 0,882 | | 0,211 | 3,693 | *0,864* | 0,878 | 0,419 | 1,840 | *0,731* | 2,012 | 1,163 | 3,480 | *<0,05* |
|  |  | Familiarity | 0,871 | 0,517 | 1,467 | *0,603* | 0,541 | | 0,232 | 1,262 | *0,155* | 0,538 | 0,312 | 0,929 | *<0,05* | 0,817 | 0,526 | 1,270 | *0,370* |
|  |  | T_PET | 2,458 | 0,882 | 6,849 | *0,085* | 0,938 | | 0,458 | 1,919 | *0,860* | 0,789 | 0,414 | 1,505 | *0,472* | 1,785 | 1,173 | 2,718 | *<0,05* |
|  |  | N_PET | 2,923 | 1,677 | 5,095 | *<0,001* | 4,790 | | 2,387 | 9,610 | *<0,001* | 2,236 | 1,396 | 3,582 | *<0,005* | 2,231 | 1,527 | 3,261 | *<0,001* |
|  |  | M_PET | 6,324 | 3,409 | 11,730 | *<0,001* | 20,124 | | 8,969 | 45,151 | *<0,001* | 4,362 | 2,663 | 7,144 | *<0,001* | 4,202 | 2,814 | 6,277 | *<0,001* |
|  | Multivariable | Age | 1,020 | 0,998 | 1,043 | *0,079* | 1,001 | | 0,973 | 1,030 | *0,964* | 1,026 | 1,007 | 1,046 | *<0,05* | 1,024 | 1,009 | 1,040 | *<0,005* |
|  |  | Comorbidities | 0,739 | 0,311 | 1,756 | *0,493* | 1,219 | | 0,254 | 5,859 | *0,805* | 1,027 | 0,456 | 2,310 | *0,949* | 2,338 | 1,321 | 4,138 | *<0,005* |
|  |  | Familiarity | 1,345 | 0,766 | 2,360 | *0,302* | 0,871 | | 0,361 | 2,102 | *0,758* | 0,654 | 0,365 | 1,171 | *0,153* | 0,633 | 0,397 | 1,008 | *0,054* |
|  |  | T_PET | 2,233 | 0,759 | 6,570 | *0,144* | 1,164 | | 0,516 | 2,623 | *0,715* | 0,664 | 0,342 | 1,288 | *0,226* | 1,690 | 1,086 | 2,630 | *<0,05* |
|  |  | N_PET | 2,139 | 1,163 | 3,936 | *<0,05* | 2,416 | | 0,950 | 6,149 | *0,064* | 0,958 | 0,558 | 1,643 | *0,875* | 1,553 | 1,018 | 2,369 | *<0,05* |
|  |  | M_PET | 5,620 | 2,813 | 11,229 | *<0,001* | 11,405 | | 3,854 | 33,748 | *<0,001* | 3,967 | 2,290 | 6,873 | *<0,001* | 2,910 | 1,858 | 4,558 | *<0,001* |
| OS | Univariable | Age | 1,012 | 1,001 | 1,023 | *<0,05* | 1,018 | | 0,997 | 1,039 | *0,098* | 1,014 | 0,996 | 1,032 | *0,133* | 1,034 | 1,018 | 1,050 | *<0,001* |
|  |  | Comorbidities | 1,444 | 0,964 | 2,164 | *0,075* | 1,269 | | 0,509 | 3,163 | *0,609* | 0,869 | 0,445 | 1,698 | *0,681* | 0,664 | 0,208 | 2,118 | *0,489* |
|  |  | Familiarity | 0,731 | 0,548 | 0,977 | *<0,05* | 1,000 | | 0,630 | 1,587 | *0,999* | 0,604 | 0,376 | 0,969 | *<0,05* | 1,528 | 0,999 | 2,337 | *0,051* |
|  |  | T_PET | 2,520 | 1,564 | 4,059 | *<0,001* | 0,970 | | 0,628 | 1,499 | *0,892* | 1,340 | 0,776 | 2,314 | *0,294* | 1,159 | 0,700 | 1,918 | *0,565* |
|  |  | N_PET | 0,908 | 0,698 | 1,182 | *0,474* | 0,820 | | 0,434 | 1,549 | *0,540* | 1,066 | 0,664 | 1,713 | *0,791* | 1,223 | 0,781 | 1,915 | *0,380* |
|  |  | M_PET | 0,717 | 0,295 | 1,744 | *0,463* | 1,013 | | 0,247 | 4,159 | *0,986* | 0,486 | 0,276 | 0,856 | *<0,05* | 2,905 | 1,771 | 4,764 | *<0,001* |
|  | Multivariable | Age | 1,013 | 1,001 | 1,025 | *0,039* | 1,035 | | 1,009 | 1,062 | *<0,05* | 1,006 | 0,986 | 1,026 | *0,567* | 1,034 | 1,018 | 1,051 | *<0,001* |
|  |  | Comorbidities | 1,252 | 0,822 | 1,906 | *0,295* | 1,877 | | 0,696 | 5,063 | *0,213* | 0,768 | 0,384 | 1,534 | *0,454* | 0,649 | 0,197 | 2,135 | *0,477* |
|  |  | Familiarity | 0,825 | 0,604 | 1,125 | *0,224* | 0,985 | | 0,599 | 1,621 | *0,954* | 0,570 | 0,334 | 0,975 | *<0,05* | 1,620 | 1,013 | 2,590 | *0,044* |
|  |  | T_PET | 2,180 | 1,154 | 4,118 | *<0,05* | 0,893 | | 0,532 | 1,499 | *0,668* | 1,151 | 0,643 | 2,059 | *0,636* | 2,078 | 1,195 | 3,612 | *<0,05* |
|  |  | N_PET | 0,814 | 0,610 | 1,088 | *0,164* | 0,556 | | 0,249 | 1,244 | *0,153* | 0,934 | 0,520 | 1,677 | *0,818* | 1,496 | 0,873 | 2,563 | *0,142* |
|  |  | M_PET | 0,756 | 0,274 | 2,085 | *0,589* | 0,397 | | 0,052 | 3,020 | *0,372* | 0,449 | 0,232 | 0,869 | *<0,05* | 1,813 | 1,024 | 3,210 | *<0,05* |

**Table S3**. Impact of [^18^F]FDG PET/CT on the change of management.

| **Setting** | **Change of management based on PET/CT results** | **Lymph node metastasis PET** | | | **Distant metastasis PET** | | |
| --- | --- | --- | --- | --- | --- | --- | --- |
|  |  | **-ve** | **+ve** | ***P-value*** | **-ve** | **+ve** | ***P-value*** |
| Baseline | No (235, 91.4%)  Yes (22, 8.6%) | 121 (51%)  6 (27%) | 114 (49%)  16 (73%) | *<0.05* | 226 (96%)  14 (64%) | 9 (4%)  8 (36%) | *<0.001* |
| Post-NAC | No (96, 86.5%)  Yes (15, 13.5%) | 74 (77%)  11 (73%) | 22 (23%)  4 (27%) | *NS* | 89 (93%)  9 (60%) | 7 (7%)  6 (40%) | *<0.001* |
| Recurrence | No (102, 68.8%)  Yes (42, 29.2%) | 69 (68%)  21 (50%) | 33 (32%)  21 (50%) | *<0.05* | 64 (63%)  20 (48%) | 38 (37%)  22 (52%) | *NS* |
| Advanced metastastic disease | No (127, 65.1%)  Yes (68, 34.9%) | 96 (76%)  25 (37%) | 31 (24%)  43 (63%) | *<0.001* | 87 (69%)  17 (25%) | 40 (31%)  51 (75%) | *<0.001* |

-ve=negative; +ve=positive
